# Annotations

**Published:** 1901-01-12

**Authors:** 


### ANNOTATIONS.

### The Teeth of the Nation.

IF we were a truly military nation, we certainly should give more attention to the children's teeth than we do at the present time. A committee of the British Dental Association some time ago investigated the mouths of a large number of school children, with the result that they found that 84 per cent. of those examined had carious teeth. Of course all these children were not suffering from toothache. If they had been something would have been done. The mischief is that dental caries often goes on until it has ruined the tooth for all useful purposes before any serious pain is produced, and that the children of the poor, when caries has gone on to such a stage as to cause toothache, have often nothing before them but extraction. Hence many troubles, much crowding and ill-development of the permanent teeth, and much future inability to tackle "bully beef." It is in the stage before toothache that preventive measures can best be taken, and this means that the dentist must go to the schools.

A great deal is being said just now about the necessity of providing free meals for school children, and although such a proceeding spells rank socialism, so, it must be remembered, does free education. But there is something before even food, and that is teeth. It must be remembered that there is a great hereditary and instinctive tendency among all but the very lowest type of the human race which drives parents to provide their young with food. Food of a sort then the children will be sure to have. On the other hand there is no inborn instinct which drives parents to look after their children's teeth. Yet we as a community object to nature's method of eliminating the toothless ones by starvation. So we keep these weakly ones alive sometimes in hospitals, sometimes in jails, sometimes in workhouses, but always at great expense, while the great multitude of those who are not thrown entirely upon our hands, lead a less healthy and less productive life than would be the case if they had been thrown upon the world uneducated, perhaps, but with good teeth. That the State should take care of the children's teeth is a matter not to be lightly put aside on the plea of its socialistic tendencies. It is a matter of both military and national importance.

Whether the State might not dig even deeper to the root of the matter, destroy all feeding-bottles, and punish with fine and imprisonment all mothers who do not suckle their own infants, is a still larger question which we will leave the Socialists to discuss.

### Our Fat Friends.

ARTISTIC traditions always represent John Bull as rather stout, a man who carries his weight well; Brother Jonathan on the other hand, is quite different, thin, fine drawn, and enterprising, but with no flesh to spare. Times, however, change, and with them people. From being a settler and a frontier rider Jonathan has become a city man; he has become comfortable and well-to-do, and, like other people of that ilk, is getting fat. It is very sad, but we have it on good American authority. The editor of the New York *Medical News* has of late been "positively amazed" to see how fat the well-to-do people of that great city are becoming, and with the laudable object of getting at the bottom of the matter has sent his young man out into the streets to count the protuberant abdomens which infest the Broadway. In this most representative thoroughfare he found that no less than 35 per cent. of the people counted had "excessively developed abdomens," while in "the corridors of one of the high-class residential hotels the number of similarly abnormal individuals in a total of 100 was 70." In another hotel of a poorer class he found, however, that only 11 per cent. of the people were afflicted with this "deformity," which perhaps was only natural. Looking round for the origin of these phenomena, and knowing well in what directions new-found riches tend to drag their owners, this astute American editor points to muscular laziness and over-feeding as the prime causes of the mischief. "Success or a modicum of success means three hearty meals a day, and in many cases an evening meal thrown in." Added to this moreover, there is "a state of absolute muscular laziness." People who used to walk now drive, or go by train, or jump on to an electric car. Formerly they used to go up and down stairs fifty times a day; now they either live in a flat or an apartment in an hotel, and lifts instead of the legs do all the work. And so they get fat. All this is true enough, but at the same time, in seeking the cause of the sleek adiposity which afflicts so many business men, one must remember that the muscular laziness, the heavy meals, the frequent stimulants, from which this condition springs, are themselves the result of the wear and strain of business which leaves its votaries but little time or energy for mere exercise. All the more reason, however, why it should be taken. Much of the sinking, tired, and empty feeling from which business men who work their brains alone so often suffer, is due to the accumulation of toxins in the system which want "working off." Two meals a day and active exercise are the preventative, and there is no exercise which can be got at any time and by anybody, to the extent that walking can. But to do good it must not be sauntering. Really "smart" walking is what is wanted. The one drawback is that nothing is "dull"—a curious contradiction.

### The Drinking of Methylated Spirit.

THE varieties of drinking are endless. The most innocent form is probably that in which alcohol is taken along with other kinds of food either at a meal or, as in the case of a thirsty labourer, in the form of a draught of beer, a drink of which contains a modicum of nourishment. A pint of good ale is indeed said to contain as much carbo-hydrate as one and a fifth ounces of bread, which explains the saying, common among working people, that beer is both meat and drink. The worst form probably is where alcohol is taken for its merely stimulating effect—to lift one up, to remove an inward craving, to produce a sensation of well-being—and a good deal of the evil of this form of drinking arises from the fact that alcohol is most effectual in this direction when it is strong. Herein lies much of the evil of spirit-drinking. What, then, are we to say about the drinking of a methylated spirit which we believe is now indulged in to a large extent in certain quarters? Methylated spirit is nasty, but it is very strong, and if for 5d. or 6d. one can buy a whole pint of a spirit containing nearly twice as much alcohol as whisky, the question of taste sinks into insignificance with those who crave merely for stimulation. So that they get the alcohol, they can by practice train themselves to swallow a little naphtha along with it. We are not sure that after a time the drinkers of this vile spirit do not even develop a taste which would lead them to turn aside from pure and mellow drinks. In any case the possibility of people flying to such a horrid alternative must be borne in mind by those who would exercise compulsion in regard to the ordinary alcoholic beverages.

